# First Screen-Printed Sensor (Electrochemically Activated Screen-Printed Boron-Doped Diamond Electrode) for Quantitative Determination of Rifampicin by Adsorptive Stripping Voltammetry

**DOI:** 10.3390/ma14154231

**Published:** 2021-07-29

**Authors:** Jędrzej Kozak, Katarzyna Tyszczuk-Rotko, Magdalena Wójciak, Ireneusz Sowa, Marek Rotko

**Affiliations:** 1 Faculty of Chemistry, Institute of Chemical Sciences, Maria Curie-Skłodowska University in Lublin, 20-031 Lublin, Poland; jedrekkozak@onet.pl (J.K.); marekrotko@poczta.umcs.lublin.pl (M.R.); 2 Department of Analytical Chemistry, Medical University of Lublin, 20-093 Lublin, Poland; magdalena.wojciak@umlub.pl (M.W.); i.sowa@umlub.pl (I.S.)

**Keywords:** electrochemically activated screen-printed boron-doped diamond sensor, first screen-printed sensor for rifampicin determination, differential pulse adsorptive stripping voltammetry, river water and urine samples

## Abstract

In this paper, a screen-printed boron-doped electrode (aSPBDDE) was subjected to electrochemical activation by cyclic voltammetry (CV) in 0.1 M NaOH and the response to rifampicin (RIF) oxidation was used as a testing probe. Changes in surface morphology and electrochemical behaviour of RIF before and after the electrochemical activation of SPBDDE were studied by scanning electron microscopy (SEM), CV and electrochemical impedance spectroscopy (EIS). The increase in number and size of pores in the modifier layer and reduction of charge transfer residence were likely responsible for electrochemical improvement of the analytical signal from RIF at the SPBDDE. Quantitative analysis of RIF by using differential pulse adsorptive stripping voltammetry in 0.1 mol L^−1^ solution of PBS of pH 3.0 ± 0.1 at the aSPBDDE was carried out. Using optimized conditions (E_acc_ of −0.45 V, t_acc_ of 120 s, ΔE_A_ of 150 mV, ν of 100 mV s^−1^ and t_m_ of 5 ms), the RIF peak current increased linearly with the concentration in the four ranges: 0.002–0.02, 0.02–0.2, 0.2–2.0, and 2.0–20.0 nM. The limits of detection and quantification were calculated at 0.22 and 0.73 pM. The aSPBDDE showed satisfactory repeatability, reproducibility, and selectivity towards potential interferences. The applicability of the aSPBDDE for control analysis of RIF was demonstrated using river water samples and certified reference material of bovine urine.

## 1. Introduction

Rifampicin (RIF) ((3-[[(4-methyl-1-piperazinyl)-imino]-methyl])-rifamycin) is a semi-synthetic macrocyclic antibiotic, which is a derivative of rifamycin antibiotics produced by fermentation of the strain *Streptomyces mediterranei*. Rifampicin is an odorless red powder that is very slightly soluble in water, acetone, alcohol, and ether. It is soluble in methanol and ethyl acetate, and easily soluble in chloroform [1,2]. Rifampicin is a first-line antibiotic along with isoniazid, pyrazinamide, ethambutol, and streptomycin in the treatment of pulmonary and extrapulmonary tuberculosis and has a unique role in killing semi-dormant tubercle bacilli (*Mycobacterium tuberculosis*) [3]. In a standard treatment procedure for tuberculosis, all four drugs are administered in various combinations over the first 2 months, and isoniazid and rifampicin are continued for the next 4 months [4]. RIF is also used for the treatment of leprosy, and some types of osteomyelitis and endocarditis. The action of this antibiotic is based on the inhibition of DNA-dependent RNA polymerase in bacterial cells, resulting in suspending their growth [5].

Inappropriate dosage during the long-term treatment period often leads to drug resistance and even death despite the disease being curable. Therefore, possible drug dosing irregularities are monitored. The simplest approach to detecting dosing abnormalities is to assess the patients’ urine levels of rifampicin. Therefore, many methods are used to determine rifampicin, including supercritical fluid chromatography (SFC) [2], high-performance liquid chromatography (HPLC) [3], fluorescence quenching [4], liquid chromatography-tandem mass spectrometry (LC-MS/MS) [5,6,7], ultra performance liquid chromatography (UPLC) [8], and spectrophotometry [1,9]. Electrochemical methods such as amperometry [10] and voltammetry [11,12,13] are also used here.

One of the popular electrodes working in voltammetry is a boron-doped diamond electrode (BDDE), which is an alternative to classical carbon electrodes. A diamond, a wide gap insulator, can be converted into a metal conductor with strong boron doping [14]. Most BDDEs are produced by the chemical vapor deposition technique and the properties of the electrode can be manipulated depending on the doping agent (B and C), surface termination, impurity level (sp3/sp2 ratio), morphological factors, and crystallographic orientation [15]. The BDDE provides superior chemical stability, low background current, a very wide potential window of water stability, low double-layer capacitance, chemical inertness, and long life-time [16,17]. One of the most valuable properties of BDDEs is the electrogeneration of hydroxyl radicals under polarization at high anodic potentials resulting in a low electrochemical activity for the oxygen evolution reaction and a high chemical reactivity for organics oxidation [18]. Furthermore, BDDEs are stable at extreme temperatures and pressures and resistant to fouling, so are ideal for the application of portable sensors for in situ measurements over extended periods of time, even in harsh environments [19].

The aim of the work presented here was to develop a simple, fast and highly sensitive voltammetric procedure for the determination of RIF in urine and water samples using an electrochemically activated screen-printed boron-doped diamond electrode (aSPBDDE). It should be noted that for the first time rifampicin was determined using a screen-printed sensor.

## 2. Materials and Methods

### 2.1. Apparatus

Voltammetric experiments were undertaken using a µAutolab analyzer (Eco Chemie, Utrecht, The Netherlands) controlled by GPES 4.9 software. The measurements were performed in a classic electrochemical cell with a commercially available screen-printed sensor (Metrohm-DropSens, Oviedo, Spain). The same analyzer controlled by FRA 4.9 software was also used to record Nyquist plots in the electrochemical impedance spectroscopy (EIS) method. The three-electrode sensor consisted of a boron-doped diamond (BDD) working electrode, a carbon auxiliary electrode, and a silver pseudo-reference electrode. In order to characterize the aSPBDDE, the optical profiles and the microscopic images of the sensors were recorded using a Contour GT-K1 optical profilometer (Veeco, New York, NY, USA) and a high-resolution scanning electron microscope Quanta 3D FEG (FEI, Hillsboro, OR, USA). The optical profiles were obtained using vertical scanning interferometry (VSI) mode with magnification of 40×. The SEM experiments were carried out under conditions (acceleration voltage of 5.0 kV, horizontal field width of 5.97 µm, working distance of 9.8 mm, magnification of 25,000×). Chromatographic measurements were performed on a VWR Hitachi Elite LaChrom HPLC system equipped with a spectrophotometric detector (PDA), an XB-C18 reversed phase core-shell column (Kinetex, Phenomenex, Aschaffenburg, Germany) (25 cm × 4.6 mm i.d., 5 μm), and EZChrom Elite software (version 3.2 SP2, Merck, Germany).

### 2.2. Reagents and Solutions

Rifampicin (Sigma-Aldrich, Darmstadt, Germany) was dissolved in ethanol to prepare 1.0 mM stock solution. This solution was diluted in 0.1 M phosphate buffer saline (PBS) with a pH value of 7.5 ± 0.1. The dilutions were prepared each day. The effect of the pH of the supporting electrolyte on the RIF signal was investigated using 0.1 mol L^−1^ PBS solutions with a pH value of: 3.0 ± 0.1, 4.5 ± 0.1, 6.0 ± 0.1, 7.5 ± 0.1, 8.5 ± 0.1, 9.5 ± 0.1, and 11.0 ± 0.1. The effect of inorganic interferences was examined using standard solutions (Merck) of: Mg(II), Ca(II), Cu(II), Cd(II), Pb(II), Ni(II), Fe(III), and V(V). The influence of organic substances was checked for reagents purchased from Sigma-Aldrich: glucose, ascorbic acid, dopamine, epinephrine, uric acid, acetylsalicylic acid, amoxicillin, and from Fluka—Triton X-100. Acetonitrile and trifluoroacetic acids (TFA) were HPLC-grade (Merck, Darmstadt, Germany). All solutions were prepared using ultrapurified water (>18 MW cm, Milli-Q system, Millipore, UK).

### 2.3. Preparation of Activated Screen-Printed Boron-Doped Diamond Electrode (aSPBDDE)

Before each series of measurements (after each solution change in the electrochemical cell), the SPBDDE was electrochemically activated. The activation consisted of five voltammetric cycles between 0 and 2 V at a scan rate of 100 mV s^−1^ in a solution of NaOH at a concentration of 0.1 M. After activation, the sensor was rinsed with deionized water and used for RIF determination.

### 2.4. Rifampicin (RIF) Differential Pulse Adsorptive Stripping Voltammetric (DPAdSV) Analysis

Voltammetric analysis of RIF under optimized conditions were carried out in 0.1 M solution of PBS (pH of 3.0 ± 0.1). An accumulation potential (E_acc_) of −0.45 V was applied during stirring for 120 s (accumulation time—t_acc_). The differential pulse adsorptive stripping voltammetric (DPAdSV) curves were recorded in the potential range from −0.25 to 1 V with an amplitude (ΔE_A_) of 150 mV, a scan rate (ν) of 100 mV s^−1^, and a modulation time (t_m_) of 5 ms. The background curve was subtracted from each voltammogram. The average values of Ip are shown with the standard deviation of n = 3.

### 2.5. RIF High-Performance Liquid Chromatography (HPLC)/PDA Analysis

HPLC conditions were based on the literature [20]. Separation was achieved using a mixture of acetonitrile and water with 0.025% of trifluoroacetic acid (50:50, *v*/*v*) as the mobile phase. The flow rate was 1.0 mL min^−1^ and temperature was set at 25 °C. The injection volume was 20 µL. All samples were analysed in triplicate at a wavelength of 330 nm. Quantification was performed using the calibration curve constructed based on peak areas of standard solutions of RIF.

## 3. Results and Discussion

### 3.1. Characteristics of aSPBDDE Sensors

**Figure 1:**
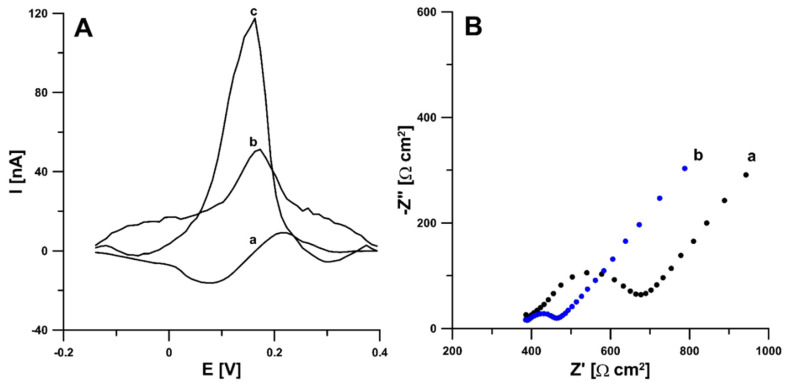
(**A**) Voltammograms of 0.2 nM rifampicin (RIF) in 0.1 M phosphate buffer saline (PBS) of pH 7.5 obtained at the bare screen-printed boron-doped diamond electrode (SPBDDE) (a), electrochemically activated in 0.1 M acetate buffer of pH 4.0 containing 10 mM H_2_O_2_ SPBDDE (b) and electrochemically activated in 0.1 M NaOH SPBDDE (c). The differential pulse adsorptive stripping voltammetric (DPAdSV) parameters: Eacc of −0.25 V, tacc of 60 s, ΔE_A_ of 50 mV, ν of 100 mV s^−1^ and t_m_ of 10 ms. (**B**) Nyquist plots of SPBDDE (a) and aSPBDDE (b).

**Figure 2:**
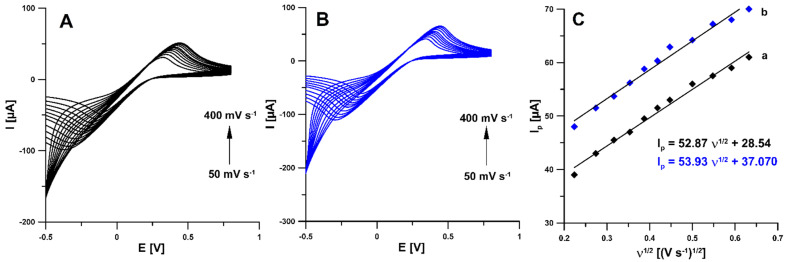
Cyclic voltammograms recorded in a solution of 0.1 M KCl containing 5.0 mM K_3_[Fe(CN)_6_] at the bare SPBDDE (**A**) and electrochemically activated in 0.1 M NaOH SPBDDE (**B**). (**C**) Dependence between anodic peak currents and the square root of the scan rates for the bare SPBDDE (a) and aSPBDDE (b), ν range of 50–400 mV s^−1^.

According to the literature data, activation can functionalize the electrode surface, increase the active surface, or remove surface contamination [21,22]. Therefore, in the first stage of the research, electrochemical activation (five voltammetric cycles between 0 and 2 V at a scan rate of 100 mV s^−1^) in two different solutions (0.1 M NaOH and 0.1 M acetate buffer of pH 4.0 containing 10 mmol L^−1^ H_2_O_2_) was applied. The studies showed that electrochemical activation of the electrode contributes to a significant increase in the RIF peak current. The signals obtained showed that the activation with NaOH was much more effective (Figure 1A). In order to test the influence of activation on the electrochemical properties of the electrodes, measurements were performed using cyclic voltammetry (CV) and electrochemical impedance spectroscopy (EIS). Impedance spectra (Nyquist plots) were recorded in the frequency range from 1 MHz to 0.1 Hz at a potential of 0.2 V, from a solution of 0.1 M KCl containing 5.0 mM K_3_[Fe(CN)_6_]. As can be seen in Figure 1B, electrochemical activation of the electrode (blue curve) significantly reduces the charge transfer resistance (R_ct_) compared to the unactivated electrode (black curve) (105.4 vs. 286.5 Ω cm^2^). The electrochemical activation of the SPBDDE changes the surface morphology, reduces the R_ct_ but does not change the active surface areas (A_s_) of the SPBDDE and aSPBDDE, which were 0.0146 ± 0.000510 and 0.0157 ± 0.000470 cm^2^, respectively. The active surface areas were calculated using CV measurements in 0.1 M solution of KCl containing 5.0 mM K_3_[Fe(CN)_6_] based on the Randles–Sevcik equation [23] and the dependence between anodic peak currents and the square root of the scan rates (Figure 2).

**Figure 3:**
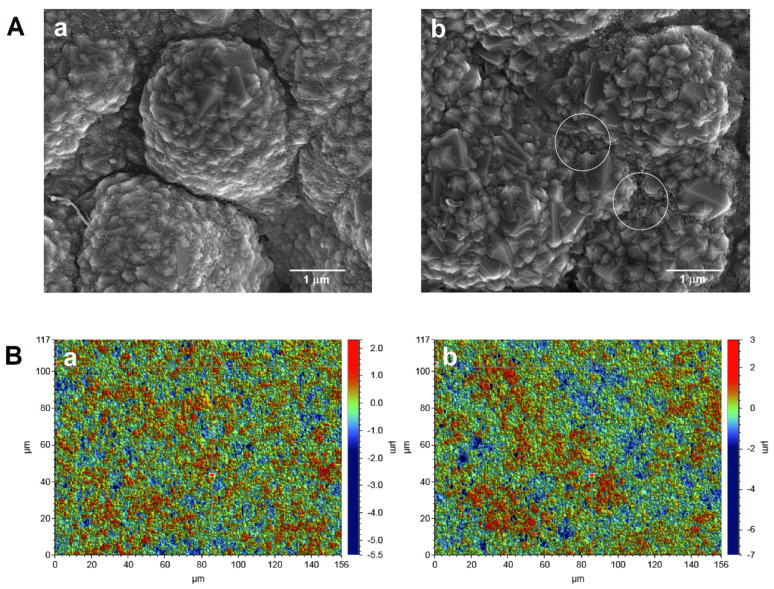
(**A**) Scanning electron microscope (SEM) images and (**B**) optical profiles of SPBDDE (a) and aSPBDDE (b).

The surface morphology of the bare SPBDDE and the electrochemically activated electrode (aSPBDDE) was examined using scanning electron microscopy (SEM) and optical profilometry. It was found that electrochemical activation causes visible changes in the surface of the working electrode, increasing the number and size of pores in the modifier layer located near the support surface (Figure 3A). This is related to the removal of organic binders existing on the electrode surface [21]. Changes in the structure of the electrode surface after activation were also found using optical profilometry. The examination of the electrodes using an optical profilometer showed an increase in surface roughness and total height of the profile (R_a_: 0.451 and 0.517 µm, and R_t_: 7.833 and 10.627 µm for the SPBDDE and the aSPBDDE, respectively) (Figure 3B).

### 3.2. Influence of pH and Concentration of Supporting Electrolyte

**Figure 4:**
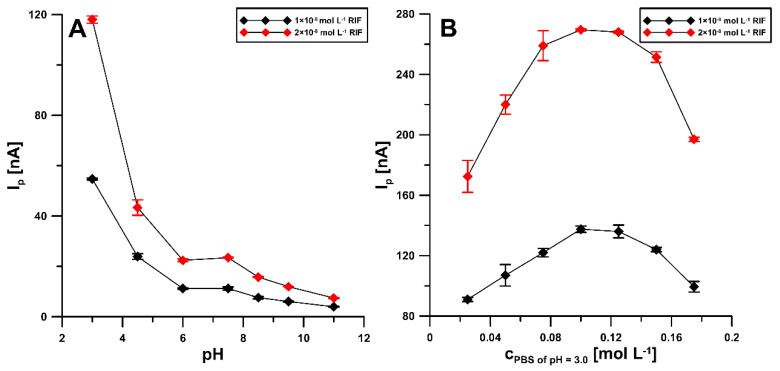
Influence of pH value (**A**) and concentration of the PBS solution of pH 3.0 ± 0.1 (**B**) on RIF peak current. The DPAdSV parameters: Eacc of −0.25 V, tacc of 60 s, ΔE_A_ of 50 mV, ν of 100 mV s^−1^ and t_m_ of 10 ms.

In order to select the optimal pH of the base electrolyte, the electrochemical behavior of 0.1 and 0.2 nM RIF in 0.1 M PBS was examined over a pH range of 3.0 to 11.0 (Figure 4A). It was observed that, with increasing pH, the peak potential shifts to less positive potential values. The maximum RIF peak current was observed at pH of 3.0 ± 0.1 and this value was considered suitable for further studies. Moreover, the influence of PBS concentration ranging from 0.025 to 0.175 M was checked (Figure 4B). The highest analytical signal of RIF was obtained for 0.1 and 0.125 M PBS and, therefore, finally the concentration of 0.1 M PBS pH 3.0 ± 0.1 was considered optimal.

### 3.3. Cyclic Voltammetry (CV) Behaviour of RIF

**Figure 5:**
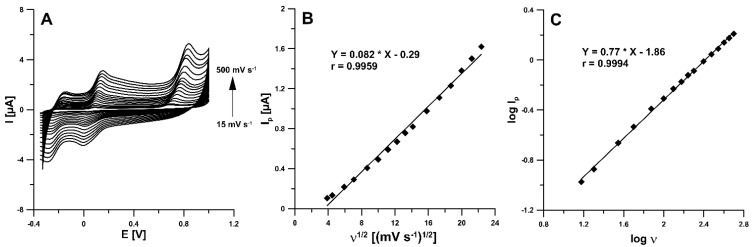
(**A**) Cyclic voltammograms recorded in 0.1 M solution of PBS (pH 3.0 ± 0.1) containing 5.0 µM RIF at different scan rates, (**B**) the dependence between Ip and ν^1/2^, (**C**) dependence between log Ip and log ν for ν from 15 to 500 mV s^−1^.

The electrochemical behaviour of RIF was examined at the aSPBDDE in the 0.1 M solution of PBS (pH of 3.0) containing 5.0 µM RIF using cyclic voltammetry and the recorded voltammograms are depicted in Figure 5A. As can be seen, RIF was oxidized quasi-reversibly. The partially reversible oxidation of RIF also occurs in voltammetric procedures using a glassy carbon electrode modified with a gold nanoparticles/poly-melamine nanocomposite [24] and a carbon paste electrode [25]. In the potential range used, three anode peaks at potentials about −0.17, 0.10 and 0.75 V and two cathode peaks at potentials about 0.02 and −0.25 V were visible (ν = 100 mV s^−1^). Two protons and two electrons are involved in the oxidation of RIF to RIF-quinone [24]. Taking into account the peak current and signal repeatability, the second oxidation RIF peak at potential about 0.10 V was selected for studies. On the basis of the obtained values of the RIF oxidation peak currents for the different scan rates from 15 to 500 mV s^−1^, the relationship between the peak current (Ip) and the square root of scan rate (ν^1/2^) indicated that the oxidation processes of RIF are controlled by diffusion at the aSPBDDE (Figure 5B). Moreover, the relationship between the log of the peak current (log Ip) and the log of the scan rate (log ν) was plotted (Figure 5C). The slope of 0.77 observed in the plot of log Ip vs. log ν indicated that this process was not purely diffusion- or adsorption-controlled.

### 3.4. Optimization of DPAdSV Parameters

**Figure 6:**
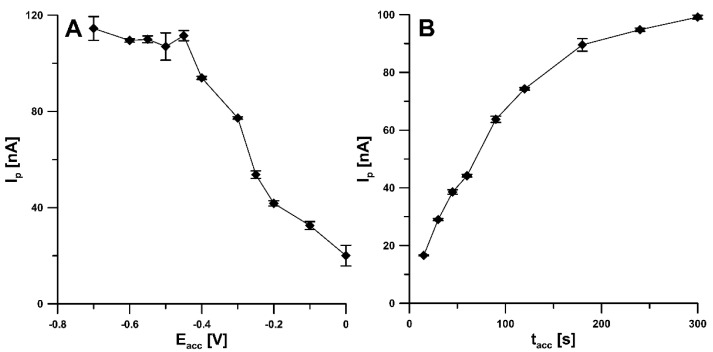
Influence of Eacc (**A**) and tacc (**B**) on the analytical signal of 1.0 and 0.5 nM RIF, respectively.

In order to obtain the best analytical signal of RIF, the effect of various parameters, including accumulation potential (Eacc) and time (tacc), amplitude (ΔE_A_), scan rate (ν), and modulation time (t_m_), on the RIF peak current was investigated. The influence of Eacc on the RIF peak current was examined in the range from 0 to −0.7 V with the tacc of 60 s. The peak current increased strongly, reaching a maximum at a potential of −0.45 V. As the potential was shifted towards more negative values, the peak current remained almost constant, hence a potential of −0.45 V was chosen as the optimal RIF accumulation potential (Figure 6A). For a potential of −0.45 V, the effect of accumulation time in the range of 15–300 s was investigated. As can be seen in Figure 6B, taking into account the highest peak currents of RIF, the t_acc_ of 300 s can be considered as an optimum. However, in order to reduce analysis time, the t_acc_ of 120 s was selected for further experiments.

**Figure 7:**
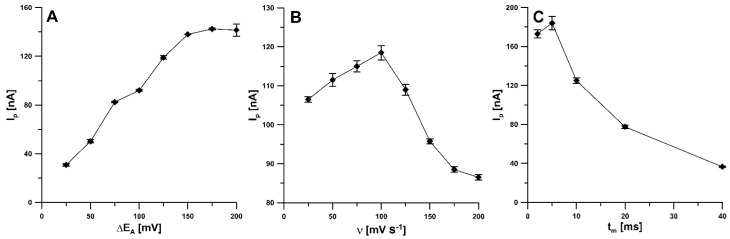
Effect of ΔE_A_ (**A**), ν (**B**) and t_m_ (**C**) on analytical response of 0.5 nM RIF. The DPAdSV parameters: E_acc_ of −0.45 V and t_acc_ of 120 s.

For ν of 100 mV s^−1^ and t_m_ of 10 ms, the amplitude was varied from 25 to 200 mV. The highest RIF signal was obtained at an amplitude value of 150 mV (Figure 7A). Then, the effect of the scan rate, ranging from 25 to 200 mV s^−1^ (ΔE_A_ of 150 mV, t_m_ of 10 ms), was tested. The RIF peak current reached its maximum value at a scan rate of 100 mV s^−1^, while a further increase in the scan rate resulted in a significant decrease in peak current. A scan rate of 100 was found to be optimal (Figure 7B). In addition, the modulation time was varied from 2 to 40 ms (ΔE_A_ of 150 mV, ν of 100 mV s^−1^). For t_m_ of 5 ms, the highest RIF signal was obtained (Figure 7C).

### 3.5. Interference Studies

**Figure 8:**
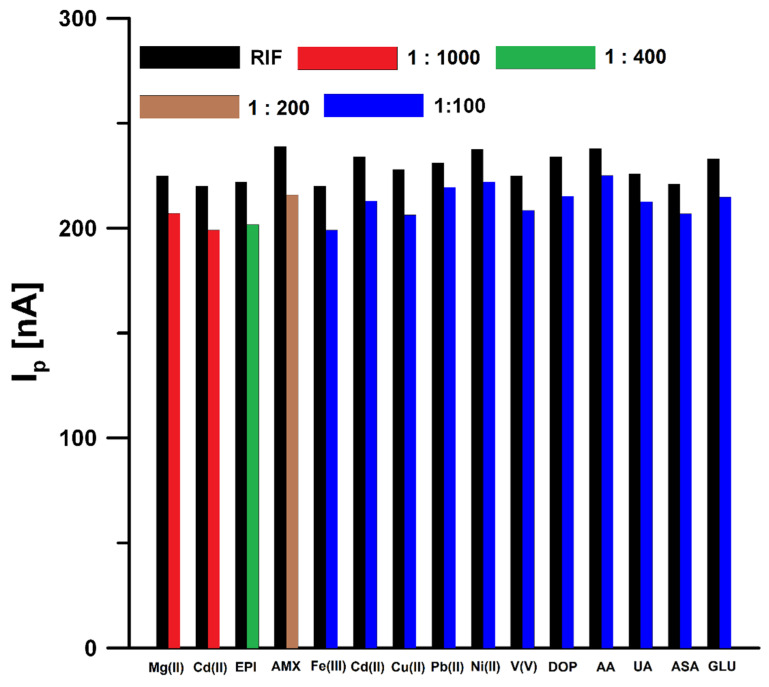
Histogram bars of the RIF peak current in the presence of interferents.

In order to test the selectivity of the proposed sensor, the influence of interferents potentially occurring in natural waters and biological fluids on the RIF voltammetric response was tested (Figure 8). The tolerance limit was defined as the concentration that gave an error of ≤10% in the determination of 0.5 nM RIF. The results obtained showed that Mg(II) (up to 1000-fold excess), Ca(II) (up to 1000-fold excess), epinephrine (EPI, up to 400-fold excess), amoxicillin (AMX, up to 200-fold excess), Fe(III) (up to 100-fold excess), Cd(II) (up to 100-fold excess), Cu(II) (up to 100-fold excess), Pb(II) (up to 100-fold excess), Ni(II) (up to 100-fold excess), V(V) (up to 100-fold excess), dopamine (DOP, up to 100-fold excess), ascorbic acid (AA, up to 100-fold excess), uric acid (UA, up to 100-fold excess), acetylsalicylic acid (ASA, up to 100-fold excess), and glucose (GLU, up to 100-fold excess) had negligible effects on the assay of RIF. Natural waters contain surfactants with a surface active effect comparable to Triton X-100 in a concentration of 0.2 to 2 ppm [26]. For this reason, the DPAdSV response of 0.5 nM RIF in the presence of 2 ppm Triton X-100 was checked and it was found that the peak current did not change by more than ±10%. Interference studies showed that the developed procedure is characterized by satisfactory selectivity and can be used for the determination of RIF in natural water samples and biological fluids without a complicated sample preparation step.

### 3.6. Analytical Characteristic

**Figure 9:**
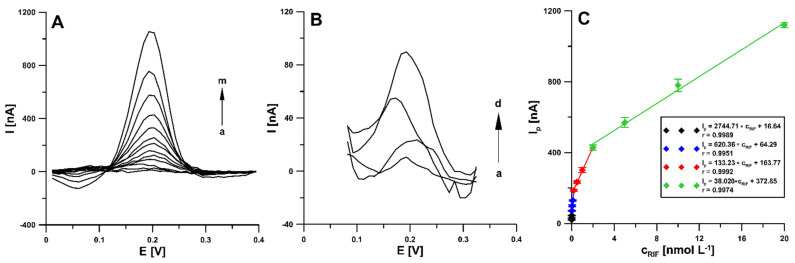
(**A**) DPAdSV curves recorded at the aSPBDDE in the PBS solution of pH 3.0 ± 0.1 containing increasing concentrations of RIF: (a) 0.002, (b) 0.005, (c) 0.01, (d) 0.02, (e) 0.05, (f) 0.1, (g) 0.2, (h) 0.5, (i) 1.0, (j) 2.0, (k) 5.0, (l) 10.0, (m) 20.0 nM. (**B**) DPAdSV curves for the RIF concentration: (a) 0.002, (b) 0.005, (c) 0.01, (d) 0.02 nM. (**C**) Calibration plot of RIF. The DPAdSV parameters: Eacc of −0.45 V, tacc of 120 s, ΔE_A_ of 150 mV, ν of 100 mV s^−1^ and t_m_ of 5 ms.

Under optimized conditions, RIF was determined in the concentration range of 2 pmol L^−1^–20 nmol L^−1^ using differential pulse adsorptive stripping voltammetry (DPAdSV). It was found that the RIF peak current increased linearly with the concentration in the four ranges: 0.002–0.02 nM (I_p_ [nA] = 2744.71 ± 71.52 × c_RIF_ [nM] + 16.64 ± 0.20), 0.02–0.2 nM (I_p_ [nA] = 620.36 ± 21.52 × c_RIF_ [nM] + 64.29 ± 2.79), 0.2–2.0 nM (I_p_ [nA] = 133.23 ± 8.54 × c_RIF_ [nM] + 163.77 ± 6.52), and 2.0–20.0 nM (I_p_ [nA] = 38.020 ± 1.14 × c_RIF_ [nM] + 372.85 ± 19.54). Figure 9 shows the voltammograms and linear ranges of the rifampicin calibration plots at the aSPBDDE. The limits of detection (LOD) and quantification (LOQ) were calculated at 0.22 and 0.73 pM, respectively, according to the definitions of LOD = 3SDa/b and LOQ = 10SDa/b (SDa—standard deviation of intercept (n = 3); b—slope of calibration curve) [27].

Table 1 presents a comparison of the different methods of RIF determination. It should be clearly stated that the developed voltammetric procedure for the determination of rifampicin enables the achievement of a much lower limit of detection than other methods [1,3,4,5,8,10]. When it comes to voltammetry, there are only three procedures in the literature in which the detection limit was lower than in this paper [28,29,30]. However, these procedures require time-consuming preparation of working electrodes, which consists of complex, multi-step modifier synthesis processes. Moreover, it is particularly noteworthy that in this work a screen-printed sensor was used for the first time to quantify RIF.

Moreover, repeatability was checked for the determination of 0.1 nM RIF and a relative standard deviation (RSD) of 2.5% (n = 10) was obtained. This RSD value proves the good repeatability of the RIF analytical signal at the aSPBDDE. The reproducibility was assessed on the basis of measurements made during the determination of 0.05 nM RIF at three sensors. The RSD value was 5.2%, which confirmed the acceptable reproducibility of the sSPBDDE.

### 3.7. Real Sample Analysis

**Figure 10:**
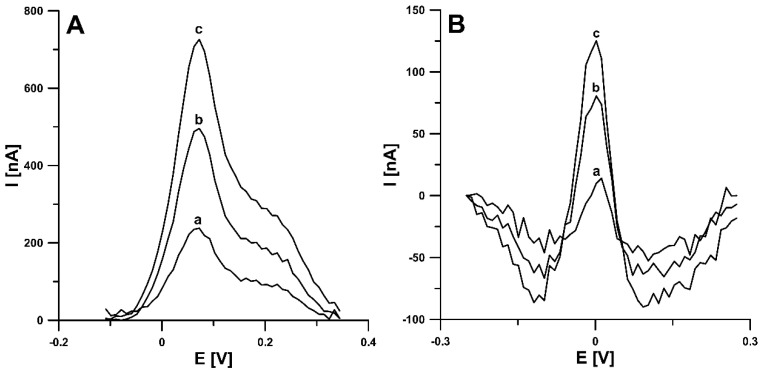
DPAdSV curves obtained for the determination of RIF in Bystrzyca river water sample (**A**): (a) 1 mL of sample + 0.1, (b) as (a) + 0.1, (c) as (a) + 0.2 nM RIF and bovine urine sample (**B**): 1 µL of sample + 0.1, (b) as (a) + 0.1, (c) as (a) + 0.2 nM RIF. The DPAdSV parameters: Eacc of −0.45 V, tacc of 30 s, ΔE_A_ of 150 mV, ν of 100 mV s^−1^ and t_m_ of 5 ms.

In order to check the usefulness of the developed RIF determination DPAdSV procedure using the aSPBDDE, the analysis of the Bystrzyca river (Lublin, Poland) and bovine urine (certified reference material, ERM-BB386, Sigma-Aldrich) samples was performed. The measurements were done by voltammetric and chromatographic method (HPLC/PDA). The results are shown in Table 2. After collecting samples from the river, they were filtered using 0.45 µm Millipore filter and stored in the refrigerator. River water samples spiked with 0.001 and 0.05 µM RIF and bovine urine samples spiked with 1.0 and 50.0 µM were analysed by the standard addition method. It should be added that to reduce interference from the sample matrix, the accumulation step was shortened to 30 s. According to our knowledge, there is no information in the literature on the concentration of RIF in environmental water samples but the concentration in urine is given. The average concentration of RIF in the urine of patients treated with this antibiotic is in the range of 55.0–67.0 µM [4]. The very low detection and quantification limits of the DPAdSV procedure (0.22 pM) obtained and a high concentration of RIF in urine samples allow for multiple dilution of the sample in the electrolyte solution, which contributes to minimizing the interference from the sample matrix. A 10-fold dilution of river water samples and 10,000-fold dilution of bovine urine samples were used for voltammetric measurements. The recovery values attained by the proposed voltammetric procedure were between 91.4% and 98.6% and indicate satisfactory accuracy of the method. Moreover, as can be seen in Table 2, no significant difference was observed between the concentrations of RIF determined by the DPAdSV at the aSPBDDE and the HPLC/PDA (the relative error values are 3.0% for Bystrzyca river samples and 3.6% for bovine urine samples). It should be added that the comparison of the results could only be done for higher RIF concentrations (river water sample spiked with 0.05 µM and bovine urine sample spiked with 50.0 × 10^−5^ µM), whereas for lower ones (0.001 and 1.0 µM), HPLC/PDA determinations were outside the LOD method. The DPAdSV curves obtained during the determination of RIF in Bystrzyca river water and bovine urine samples are shown in Figure 10.

## 4. Conclusions

In summary, in this study a simple, fast, and cost-effective differential pulse adsorptive stripping voltammetric procedure (DPAdSV) using an electrochemically activated screen-printed boron-doped diamond electrode (aSPBDDE) for quantification of rifampicin (RIF) was developed. For the first time, a screen-printed sensor was introduced for analysis of RIF. The electrochemical activation of the electrode surface using cyclic voltammetry (CV) in 0.1 mol L^−1^ NaOH resulted in changes in its morphology and a decrease in the charge transfer resistance, which translated into a significant increase in the RIF oxidation peak current. Moreover, the electrochemical behaviour of RIF in 0.1 M PBS (pH of 3.0 ± 0.1) was characterized by CV. The results obtained show that the oxidation of RIF at the aSPBDDE was not purely diffusion- or adsorption-controlled. The DPAdSV procedure developed using the aSPBDDE showed good selectivity and sensitivity. The calculated LOD and LOQ values were 0.22 and 0.73 pM, respectively. The DPAdSV procedure at the aSPBBDE was successfully used for the determination of RIF in river water and bovine urine samples. The recovery values (91.4% and 98.6%) and the good agreement with the results obtained by the DPAdSV procedure and by the referenced HPLC/PDA method (relative errors of 3.6 and 4.0%) showed satisfactory accuracy of the method. The results obtained revealed the analytical usefulness of the presented voltammetric procedure for RIF analysis in body fluids and natural water samples.

## Figures and Tables

**Table 1:** Comparison of different methods for RIF determination.

Method	Linear Range (µM)	LOD (µM)	Application	Ref.
Spectrophotometry	6.08–60.80	4.25	Pharmaceutical formulations	[1]
Fluorescence quenching	0.61–1000.0	0.085	Human urine	[4]
HPLC	0–2.0	5.86	Herbal extracts, liver microsomes	[3]
LC-MS/MS	0.030–7.78	0.30	Human plasma	[5]
UPLC	0.0790–31.60	-	Human plasma	[8]
Amperometry	-	1.69	Pharmaceutical formulations, urine	[10]
DPAdSV	0.0000020–0.020	0.00000022	Bovine urine, river water	This work

**Table 2:** The results of RIF determination in river water and bovine urine samples.

		RIF Concentration (µM) ± SD (n = 3)				
Sample	Added	Found DPAdSV	RIF Concentration Found in Electrochemical Cell	Found HPLC/PDA	Recovery * (%)	Relative Error ** (%)
Bystrzyca river	0.001 0.05	0.000973 ± 0.0000240 0.0457 ± 0.00110	0.0000973 ± 0.00000240 0.00457 ± 0.000110	<LOD 0.0471 ± 0.00250	97.3 91.4	- 3.0
Bovine urine	1.0 50.0	0.920 ± 0.0070 49.30 ± 0.40	0.0000920 ± 0.00000070 0.00493 ± 0.000040	<LOD 47.60 ± 0.018	92.0 98.6	- 3.6

* Recovery (%) = (Found DPAdSV × 100)/Added; ** Relative error (%) = ((ǀFound HPLC/PDA − Found DPAdSVǀ)/Found HPLC/PDA) × 100.

## Data Availability

The data presented in this study are available on request from the corresponding author.
